# A Disease Control-Oriented Land Cover Land Use Map for Myanmar

**DOI:** 10.3390/data6060063

**Published:** 2021-06-13

**Authors:** Dong Chen, Varada Shevade, Allison Baer, Jiaying He, Amanda Hoffman-Hall, Qing Ying, Yao Li, Tatiana V. Loboda

**Affiliations:** 1 Department of Geographical Sciences, University of Maryland, College Park, MD 20742, USA;; 2 Department of Earth System Science, Tsinghua University, Beijing 100084, China;; 3 Department of Geosciences, Texas Tech University, Lubbock, TX 79409, USA

**Keywords:** remote sensing, public health, infectious disease, malaria, Myanmar, land cover/land use map, landsat

## Abstract

Malaria is a serious infectious disease that leads to massive casualties globally. Myanmar is a key battleground for the global fight against malaria because it is where the emergence of drug-resistant malaria parasites has been documented. Controlling the spread of malaria in Myanmar thus carries global significance, because the failure to do so would lead to devastating consequences in vast areas where malaria is prevalent in tropical/subtropical regions around the world. Thanks to its wide and consistent spatial coverage, remote sensing has become increasingly used in the public health domain. Specifically, remote sensing-based land cover/land use (LCLU) maps present a powerful tool that provides critical information on population distribution and on the potential human-vector interactions interfaces on a large spatial scale. Here, we present a 30-meter LCLU map that was created specifically for the malaria control and eradication efforts in Myanmar. This bottom-up approach can be modified and customized to other vector-borne infectious diseases in Myanmar or other Southeastern Asian countries.

## 1. Summary

Accurate accounts of land cover/land use (LCLU) in the form of LCLU maps are critical sources of information that allow stakeholders to make informed decisions. Many LCLU datasets, such as the Moderate Resolution Imaging Spectroradiometer (MODIS) MCD12 Land Cover product [1] and the National Land Cover Database (NLCD) [2], are geared towards serving general needs (i.e., land planning, forest management, etc.) and are generally created based on global algorithms (i.e., algorithms that are consistently applied across the areas of interest). However, there are situations where specific LCLU classification schemes are required and these circumstances are generally associated with the efforts that are implemented on smaller spatial scales. As a result of such specific needs, the LCLU maps produced using global top-down algorithms are likely to be insufficient for use locally. Recently, publicly available datasets and algorithms designated to specific LCLU classes, such as croplands and water bodies, have been increasingly offered with a high readiness for scientific usage. As these datasets and algorithms are designed for specific purposes and are often associated with uncertainty metrics (allowing the mapping results to be adjusted locally), they are likely to be more reliable in representing the LCLU classes of interest than the datasets that are derived using top-down methods. Leveraging such datasets and algorithms that utilize local knowledge about the issues in question could be the key to the development of bottom-up LCLU maps that serve various special and localized needs.

LCLU patterns have been shown to be important for disease epidemiology and can be conveniently determined using remote sensing data. Land cover determines habitat suitability for hosts and vectors [3] and remote sensing data are commonly used to determine vector/host animal habitats. Disease risk variation, however, is not only determined by the land cover but also by the land use, which helps to understand the places that people visit and use for certain activities or during certain times of the day or year [4], and contributes to the determination of human distribution and the contact between humans and vectors [3]. LCLU maps and landscape metrics that have been derived using remote sensing have been used to understand the patterns and transmission of several diseases, including malaria, Lyme disease, cholera, dengue, Zika, etc. [5–10]. Most of such studies have used the mapped vegetation derived from remote sensing to understand the factors associated with vector presence and modeled human transmission risk, relying on the fact that most vector-borne disease transmission is dependent partly on vegetation characteristics [5]. For example, some studies have used LCLU maps at multiple spatial resolutions to predict the disease transmission risk or to study the role of LCLU in disease transmission based on the links between vector habitat preferences, LCLU classes, and disease incidence [6,10]. In contract, others have associated LCLU based landscape metrics such as edge density, patch density, and proportion of land use with disease risk [9].

The Southeastern Asian country of Myanmar, home to a population of more than 53 million people, carries one of the highest malaria burdens within the Greater Mekong region, despite having only 4% of its population [11,12]. Although Myanmar has successfully reduced the number of malaria cases by ~82% between 2012 and 2017 [13], the emergence of drug-resistant parasites in the region in recent years [14] has made Myanmar a critical battleground for the global fight against malaria. The use of satellite data and LCLU information has been demonstrated to be crucial in understanding landscape-scale malaria exposure and disease transmission. Previous work has established some linkages between the LCLU types and malaria risks. Specifically, deforestation and agricultural development have been shown to influence mosquito species abundance and malaria incidence through changes to the ecology of vector species as well as the nature and stage of the agricultural development determining patterns of human contact with vectors [7,15,16].

In addition to capturing and highlighting the areas where malaria exposure and transmission are more likely, LCLU maps could also be of great importance for malaria treatment efforts because they could provide information about the distribution of human settlements at large spatial scales. This is especially crucial for rural villages with low economic well-being and limited access to care [16,17]. However, creating LCLU maps that offer meaningful information about village distribution across the country is challenging because (1) it requires the mapping to be implemented at a 30-meter or higher resolution to ensure the successful mapping of remote villages (according to our personal observations, many remote villages in Myanmar are fairly small in extent and consist of a few buildings with small footprints), which are known to serve as the remnant pools of malaria [18], and (2) villages are quite similar to many bare ground features (e.g., riverbanks, bare mountain tops) in terms of their spectral signature. There are several existing LCLU datasets (Table 1) that provide information about human settlements that cover Myanmar at a 30-meter or higher resolution, including the SERVIR land cover dataset for the Greater Mekong region (hereafter referred to as “SERVIR Mekong land cover dataset”) [19], the Global Human Settlement Layer (GHSL) dataset [20], and the Global Human Built-up And Settlement Extent (HBASE) dataset [21]. However, due to either their multipurpose nature (SERVIR Mekong land cover) or global algorithms (GHSL and HBASE), their performance in identifying remote villages in Myanmar is limited (as shown in the Data Records section).

In this paper, we present an LCLU map of Myanmar, which is the first 30 m LCLU map designed specifically for the control and elimination of malaria in Myanmar. Being an application-oriented LCLU map, it was created following a bottom-up approach, which allows for the LCLU classes that are important for malaria transmission to be mapped with relatively higher confidence. In addition, this approach is inherently more flexible than the traditional methodologies used to produce LCLU maps, because (1) the classes and their priorities can be modified based on application-specific needs and (2) there is freedom to adopt the best available datasets and algorithms. Due to this flexibility, even though the produced LCLU map is only for the year 2016, given proper inputs, such LCLU representations could be produced for other years to develop a consistent time-series of LCLU maps (most of the classes with potentially high interannual variability in our classification scheme, such as managed forests and villages, can be updated annually). Moreover, similar LCLU maps could be developed for other vector-borne diseases based on their respective epidemiology or disease transmission characteristics. Additionally, due to its strong focus on remote villages, this methodology could potentially be used in other applications that are unrelated to public health but have equally strong demands on the accurate mapping of villages. This may include international humanitarian efforts, considering Myanmar has one of the highest refugee populations by country of origin, due to long-lasting and ongoing domestic conflicts [22,23].

## 2. Data Description

**Figure 1.**
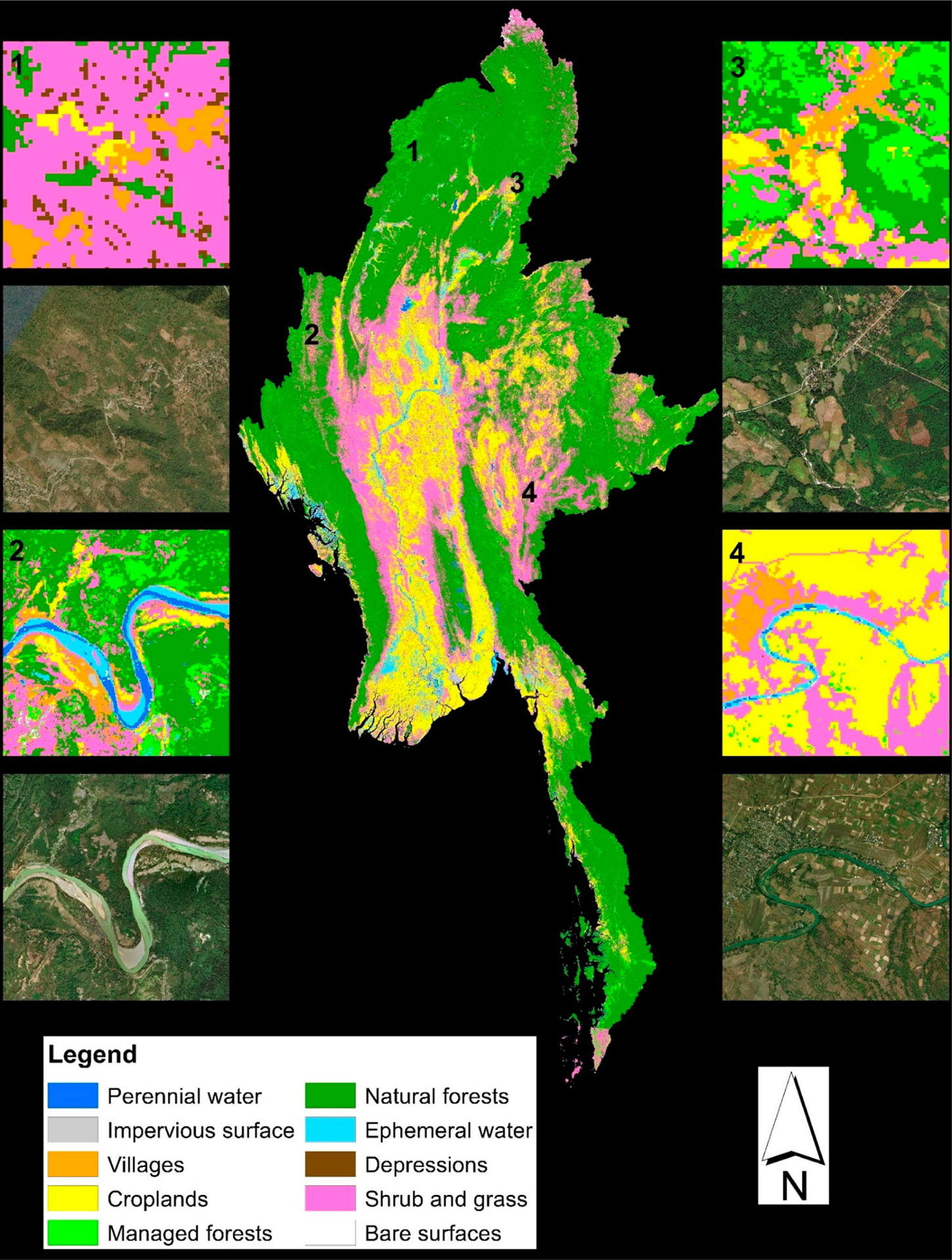
Overview of the presented LCLU map for Myanmar. Insets 1–4 shows zoomed-in views of 4 locations, respectively.

**Figure 2.**
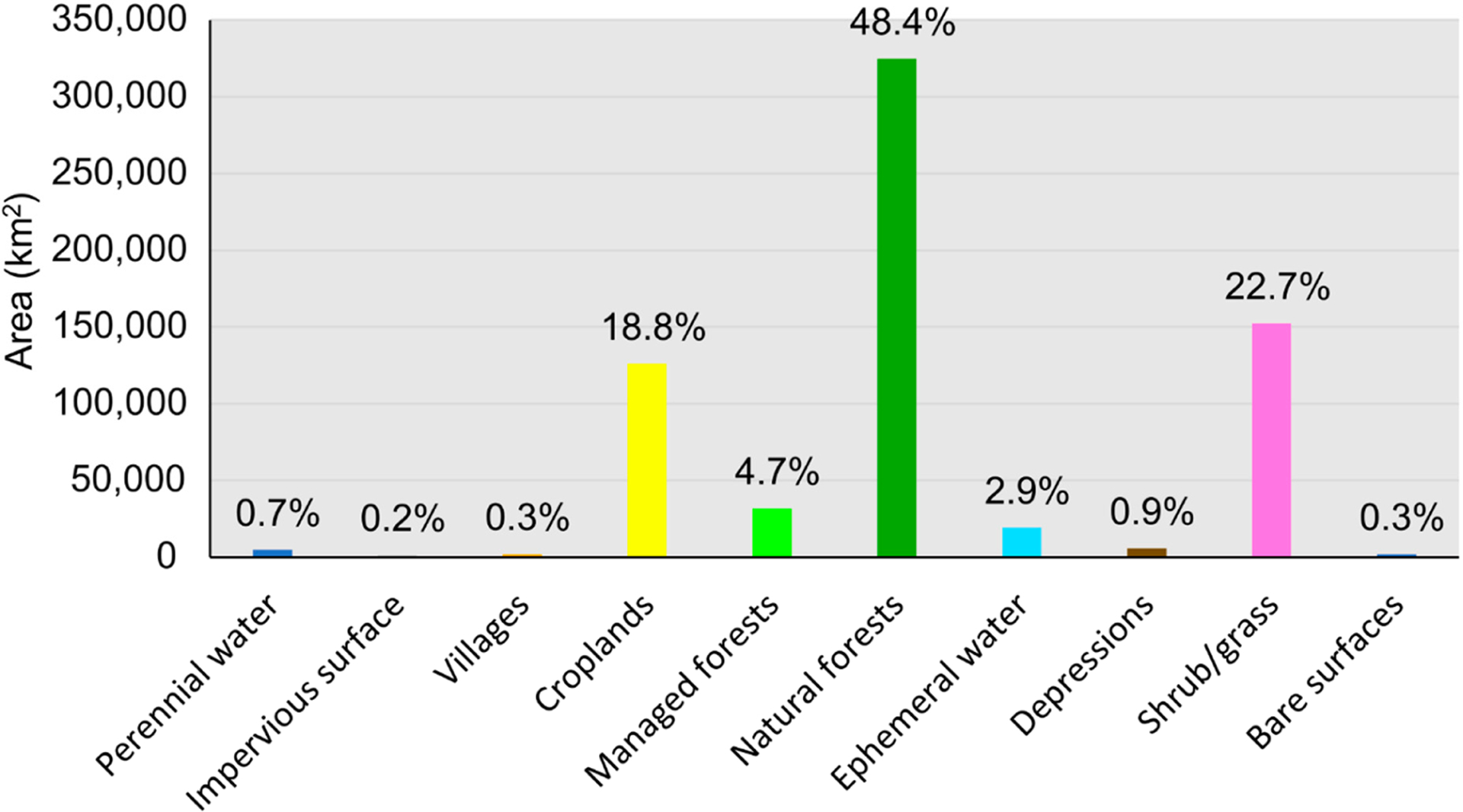
Distribution in terms of total area of the ten classes contained in the presented LCLU map.

The presented dataset (https://doi.org/10.1594/PANGAEA.921126) [24] has been released through PANGAEA (https://www.pangaea.de/), an open data repository designated for the publication of datasets related to geoscience and environmental science. The dataset contains two data files. One is the LCLU map in a single GeoTiff file, and the other is a Microsoft Excel table listing the classes contained in the LCLU map. Figure 1 shows an overview of the entire LCLU map, with zoomed-in views of four different locations across Myanmar. Figure 2 shows the distribution of the ten classes in terms of the total area as mapped by the LCLU map across Myanmar.

**Figure 3.**
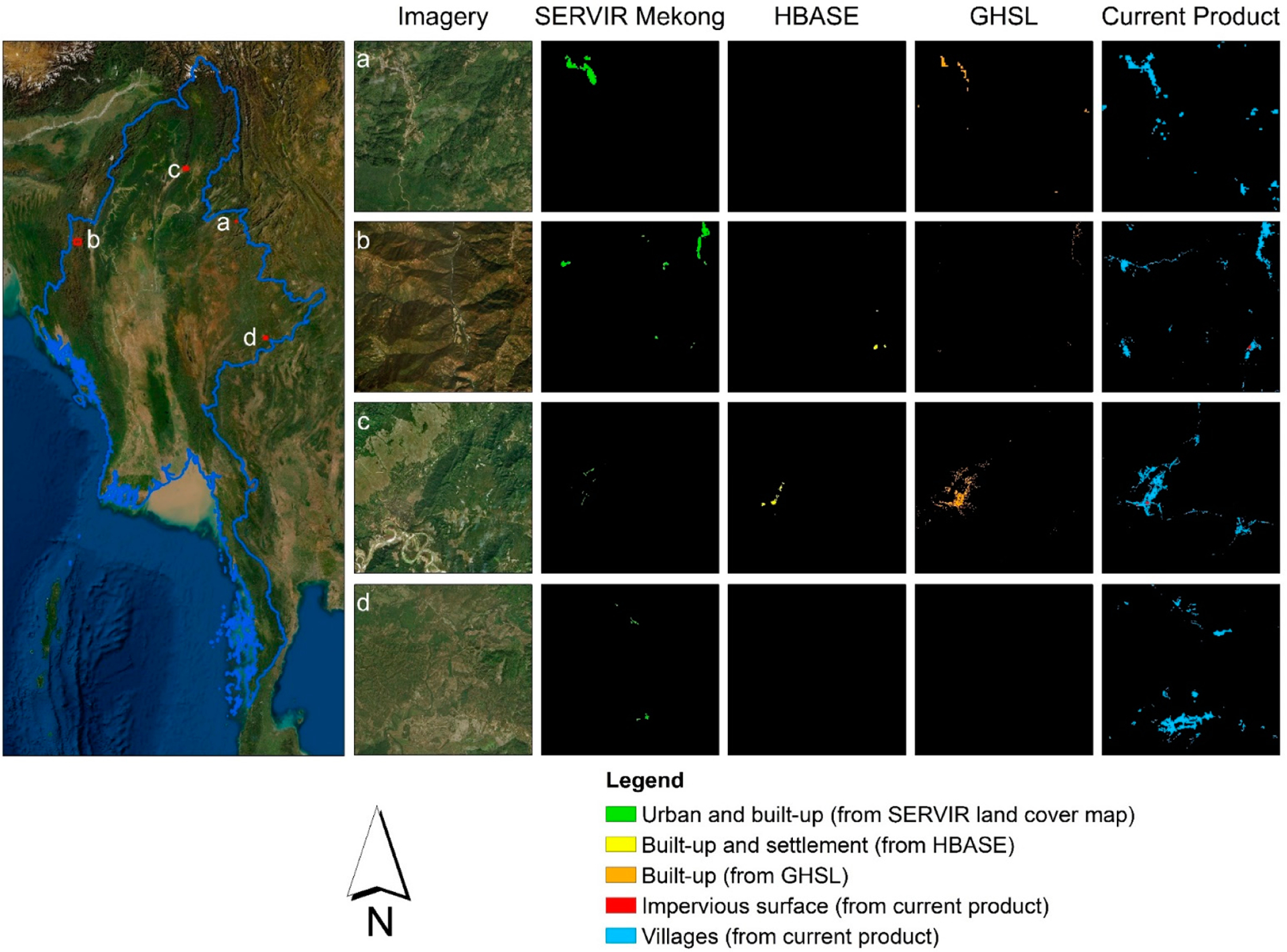
A comparison between the current product and three existing land cover products in terms of the representation of human presence (SERVIR Mekong [19]: urban and built-up; HBASE [21]: built-up and settlement; GHSL [20]: built-up; current product: impervious surface and villages). All other classes irrelevant to human presence in the compared products are masked out (indicated in black). The SERVIR Mekong land cover map that was compared and displayed was produced for 2016 (the same year as our product).

We conducted a visual comparison between our map and the following three existing 30 m datasets that contain settlement-related classes (Table 1): the SERVIR Mekong land cover dataset [19], the GHSL dataset [20], and the HBASE dataset [21]. The comparison (Figure 3) shows that our product provides a much more spatially comprehensive representation of the classes related to human presence than all of the other datasets, especially in remote rural areas, where the villages typically consist of bare ground and small individual buildings. The difference observed in the mapping of human settlements between our product and the existing products is not surprising considering we adopted a locally developed algorithm to specifically map the village class.

## 3. Methods

This LCLU map is designed to support malaria research and the operational interventions for malaria elimination in Myanmar. Hence, we designed a classification scheme to include the land cover classes associated with the habitat suitability of the malaria vector—the *Anopheles spp*. mosquito—and the land use classes that can potentially increase human exposure to malaria. Our LCLU map was developed using several published LCLU datasets, modified ancillary data (for the depression and bare surface classes), and an independently mapped class (village). The combination of data sources inevitably led to several classes overlapping at certain locations. This meant that there was a need for a mapping hierarchy to ensure that each individual pixel of the final LCLU map belonged to a single class. Therefore, the classification scheme, which is discussed in detail subsequently, describes not only the classes that are included in the map but also the hierarchy of the mapped classes.

**Figure 4.**
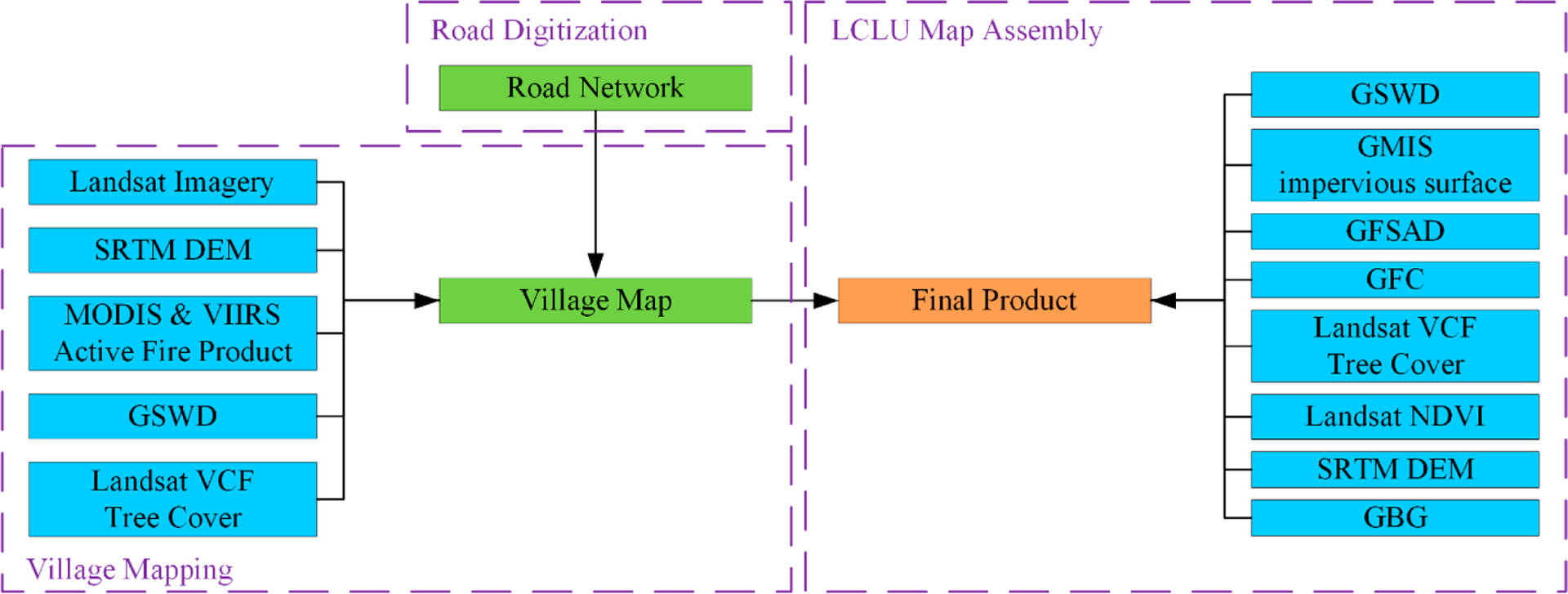
Workflow for the development of the presented dataset. The purple boxes with dashed line boundaries indicate the three major technical steps (corresponding to the subsections of Methods). The blue and green boxes indicate the major data inputs and intermediate outputs, respectively. The orange box represents the final output, which is the produced LCLU map. Abbreviations: a Shuttle Radar Topography Mission (SRTM), a digital elevation model (DEM), a Moderate Resolution Imaging Spectroradiometer (MODIS), a Visible Infrared Imaging Radiometer Suite (VIIRS), the Landsat Vegetation Continuous Fields (Landsat VCF) project [25], Very High Resolution (VHR), the global surface water dynamics (GSWD) product, the Global Man-made Impervious Surface (GMIS) product [26], the Global Food Security Analysis-Support (GFSAD) product [27], the Global Forest Change (GFC) product [25], the normalized difference vegetation index (NDVI) [28], the global bare ground gain (GBG) product [29].

The methodology of our map development can be broken down into three major steps (Figure 4). First, the roads in Myanmar were manually digitized, leading to a much more complete and up-to-date road network dataset for Myanmar compared with other existing public datasets that we were aware of. Second, the resultant road network dataset was used in conjunction with a set of publicly available satellite imagery and ancillary data or their derivatives to map the village extent in Myanmar. Finally, the LCLU map was assembled based on the mapped village extent and a series of additional datasets.

### 3.1. Road Digitization

**Figure 5.**
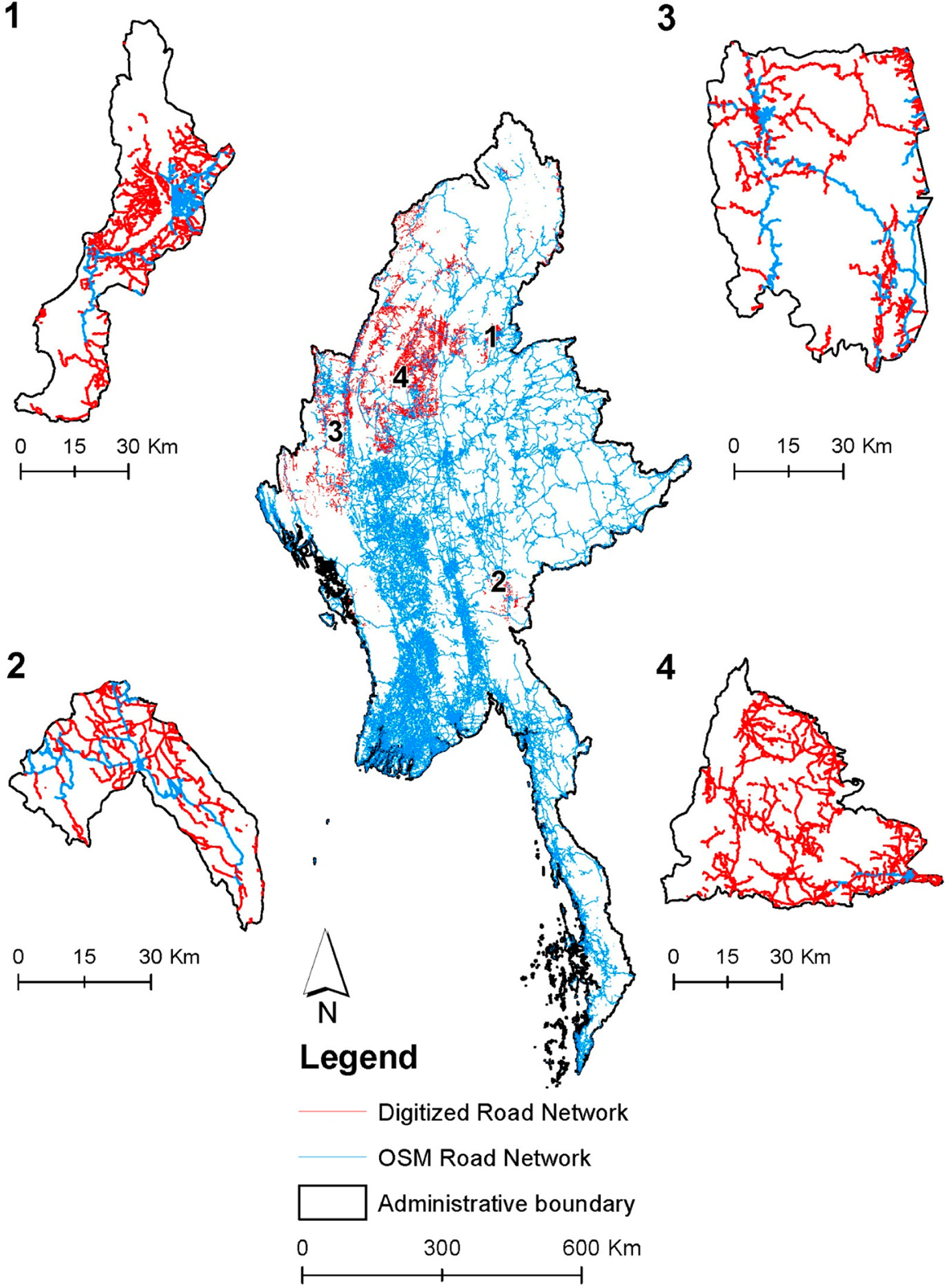
The digitized road network (in red lines) in comparison to the OpenStreetMap road network [30] (in blue lines) of Myanmar, with edits to the dataset made as necessary. Panels one through four show comparisons of the digitized road network and the OSM road network of (1) Bhamo Township in the Kachin State, (2) Demoso Township in the Kayah State, (3) Hakha Township in the Chin State, and (4) Kyunhla Township in the Sagaing Region. These four regions were selected because they were among the areas that have the highest number of roads (in distance) added and represent different parts of the country. Administrative boundaries are from MIMU.

Road networks are an important indicator for understanding population distribution and human mobility. However, after conducting a visual assessment of VHR imagery and existing road network data in Myanmar, we determined that there are limited road network data available for Myanmar, especially in mountainous regions. From April 2019, as the basis of our effort using the administrative boundaries downloaded from the Myanmar Information Management Unit (MIMU, http://geonode.themimu.info, accessed in April 2019), we digitized roads using VHR Google Earth images based on the Myanmar Roads dataset that was provided by the Humanitarian OpenStreetMap Team [30]. Notably, we have added road networks in vast areas of Myanmar (Figure 5, center) and individual townships (select areas in different parts of the country are shown in Figure 5, panels 1–4). Townships are the third-level administrative divisions below districts (level two) and states/regions/union territories (level one). We digitized over roughly 25,000 km of additional roads.

### 3.2. Village Mapping

In Myanmar, rural populations are particularly susceptible to malaria because of their relatively lower economic status and access to care [17]. Therefore, understanding the distribution of the settlements, especially those that are remote, has huge implications for developing effective interventions. It is for that reason that our LCLU map includes a village class designated primarily to rural settlements that are not characterized by impervious surfaces (hence not mapped by impervious surface datasets such as GIMS [26]). Hoffman-Hall, Loboda, Hall, Carroll, and Chen [18] developed a random forest-based village mapping algorithm for Ann, a township in the Rakhine State of Myanmar. Considering Ann is only a small part of Myanmar (0.9% of the total area of Myanmar), an improved algorithm was needed to account for the great heterogeneity in environmental, socio-demographic conditions, and spatial patterns across the country. Here, building upon the foundation proposed by Hoffman-Hall, Loboda, Hall, Carroll, and Chen [18], we developed an updated village mapping algorithm. We adopted 26 variables, which were identified by Hoffman-Hall, Loboda, Hall, Carroll, and Chen [18] to be important for village mapping. We also added a slope variable, as our preliminary analysis confirmed its influence on village distribution. Table 2 lists the total of 27 variables that were fed into our random forest models. Most of these variables were derived based on corresponding Landsat imagery, i.e., one cloud-free Landsat composite for the dry–cold season (February–April 2016) and one for the dry–hot season (November 2016–January 2017). All of the variables were processed at a spatial resolution of 30 m.

At the country level, Myanmar has large variations in terrain. Based on our field experience, the contextual relationships of the individual buildings in the villages located in the mountain regions are evidently different to those in the plains, i.e., villages are generally larger and denser in the plains, whereas in the mountains, villages are much sparser, smaller, and more irregularly shaped. Based on this information, we created two training sample pools for the village class, separating mountain and plain topography. Using the SRTM DEM data, we divided the entire country of Myanmar into two terrain classes (mountain: ≥1000 m; plain: <1000 m, using an elevation threshold we selected empirically).

We used the village point dataset published by MIMU to guide our selection of training sample points. Covering the majority of Myanmar, with more than 47,000 points, this is the most comprehensive village point dataset to our knowledge. Every village point in the MIMU dataset was buffered by 500 m (a threshold value we chose empirically), after which 2000 sample points were randomly generated with the mountain and plain areas each having 1000 points. Then, we visually examined each of the 2000 random points using VHR imagery hosted on Google Earth to see whether that point was actually placed within the village. Owing to the differences in the spatial extents of the individual villages and the inaccuracy of some of the MIMU village points, a considerable number of the random points were placed outside of villages (e.g., in croplands or forests adjacent to villages). These out-of-bound random points were preserved and used as the training sample for the “non-village” class, while those random points correctly located in villages were considered as the training sample for the “village” class.

All of the training sample points (village + non-village) were used to extract the values from the independent variables listed in Table 2, resulting in two training sample pools, one for the mountain region and one for the plain region. Each of these training sample pools was fitted to a random forest model, which was subsequently applied to the testing data (created utilizing all of the independent variables for the entire country) to identify villages across Myanmar. Additional training points for each class were added iteratively upon visual examination of classification outputs. The final training dataset consisted of 19,790 points (village: 464; non-village: 19,326) for the mountain region and 3456 points (village: 215; non-village: 3241) for the plain region (the number of training sample points for the mountain region is considerably larger than that for the plain region due to the added difficulty of characterizing villages in areas with high topographic variability). A quantitative assessment of the mapping accuracy of the village class was performed as part of the combined LCLU map.

### 3.3. LCLU Map Assembly

LCLU map classes are chosen to be representative of the landscape. Additionally, based on our understanding of the malaria distribution and spread, as well as our field knowledge of the environmental and demographic conditions in Myanmar, several classes (Table 3) are also important in the context of malaria transmission. For example, we included depressions, defined as areas where standing water periodically or sporadically emerges after rain or floods, because they are a known breeding habitat of mosquitoes [37,38]. We included croplands because they cover a substantial proportion of the landscape and, in addition to flooded paddy fields being a mosquito habitat [39], they are also an important interface where mosquito–human transmission is likely to happen, as agriculture is the main source of income for Myanmar, accounting for more than a third of its gross domestic product [40]. Similarly, managed forests were also included in our LCLU map because they usually coincide with plantations (based on our field knowledge), which are another common environment for occupation-related exposure to malaria [12,41]. The impervious surface class, characterized by larger towns/cities and large manmade structures such as roads, was included because its association with malaria distribution and spread may be different from that in villages, where living conditions and access to care are more limited.

In the end, a total of ten classes were identified for the LCLU classification scheme (Table 3). These were, subsequently, assembled from separate data sources based on the following order of priority: perennial water > impervious surface > villages > croplands > managed forests > natural forests > ephemeral water > depressions > bare surfaces > shrub/grass. The priorities were determined empirically based on the expert opinion of in-country medical researchers and public health professionals on the general knowledge regarding the relevance of the class for malaria transmission. If a pixel met the criteria of multiple classes simultaneously, it was ultimately classified into the class with the highest priority. For example, if a pixel was mapped as impervious surface by GIMS, it would be eventually merged into the impervious surface class, unless it simultaneously met the criteria for perennial water, in which case it would be merged into the perennial water class.

## 4. Validation

We assessed the map classification accuracy using a stratified random sampling design guided by the “good practices” described by Olofsson et al. [44]. We used the open-source stratified area estimator design tool available on the SEPAL platform (https://sepal.io/), a cloud-based platform developed by the United Nations Food and Agriculture Organization Forestry Department’s Open Foris team, to implement the sampling design and generate the stratified random points. The dataset was reclassified in ArcMap to exclude the depressions class. The remaining map classes, except depressions, were used as strata. The stratified area estimator design tool calculates the overall sample size and the strata sample size using the equations described by Olofsson, Foody, Herold, Stehman, Woodcock, and Wulder [44]. The expected user’s accuracies for the perennial water, villages, croplands, natural forests, and bare surfaces strata were assigned 0.75, while the impervious surface, managed forests, ephemeral water, and shrub and grass strata were assigned 0.65. The desired standard error of the map’s overall accuracy was set to 0.013. A total of 1179 samples were created with at least 75 samples per class to ensure adequate samples of rare classes [44]. The samples were buffered to 30 m × 30 m in ArcMap and reviewed against Google Earth VHR imagery.

Although the land cover classifications represent distinct categories, a combination of various LCLU classes is frequently found within a 30-meter pixel, particularly for the classes with small footprints (e.g., impervious surface), or the classes with an expected mix of different land covers (e.g., villages). Additionally, the general lack of multi-temporal VHR data creates ambiguity in the exact extent of a class at a specific point in time (the point when the class was mapped vs. when the class is assessed) particularly for highly dynamic classes (e.g., perennial or ephemeral water), but also for many other classes. To account for this potential ambiguity and mixed pixels, we adopted a linguistic scale developed by Gopal and Woodcock [45] for fuzzy accuracy assessments of classified maps. The scale ranges from 1 to 5, where (1) is “absolutely wrong”, (2) is “understandable but wrong”, (3) is “reasonable or acceptable answer”, (4) is “good answer”, and (5) is “absolutely right” [46]. An interpreter reviewed each sample against each stratum according to the five-level scale. The responses were recorded using Microsoft Access.

Two fuzzy accuracy assessment functions, MAX and RIGHT [46], were calculated to assess the map classification. The MAX function considers correctness if the interpreter properly identified the sample as a five, or “absolutely right.” The RIGHT function considers correctness if the appropriate class is identified as a three or higher, (i.e., “reasonable or acceptable answer”, “good answer”, or “absolutely right”). Overall, the weighted fuzzy accuracy of the map classification is 69.22% (Table 4).

## 5. User Notes

We would like to demonstrate two possible scenarios where our LCLU map could be used, among others.

### Scenario 1: Using our LCLU map to identify high-risk areas.

Under this scenario, it is recommended that users first assess the relationship between each of the classes of our LCLU map and the specific infectious disease in question. The identified relationships can be used to determine the weight that is assigned to each class, which ultimately can serve as the foundation of a system that highlights the high-risk areas. If interannual changes in the distribution of the high-risk areas are of interest, users can apply the algorithm outlined in this paper to the data acquired in other years and generate similar LCLU-based risk maps for other years.

### Scenario 2: Using our LCLU map to identify remote villages.

As our intercomparison shows (Figure 3), our LCLU map offers one of the highest levels of accuracy for identifying remote villages among the existing datasets. Users can integrate our map with other datasets, such as OpenStreetMap road networks and DEM, to identify villages that are isolated and might need special attention in disease monitoring and eradication. Similar to Scenario 1, users can create annual LCLU maps for multiple years based on which spatio-temporal distribution of villages can be tracked across Myanmar.

## 6. Limitation and Caveat

While our LCLU map performs well identifying villages in general (as indicated in Table 4), it is unable to capture individual buildings. In other words, standalone buildings that are not part of a perceivable settlement are likely to be misclassified into other classes. This is, however, not surprising considering that the resolution of Landsat imagery is 30 m. For the delineation of the footprints of individual buildings, VHR imagery is likely required.

We would like to point out that the performance of the cropland class is likely to be strongly subject to seasonality. This is because Myanmar has a tropical monsoon climate, which means there is great seasonality in the precipitation patterns across the country. During the monsoon season, floods are prevalent in Myanmar, which may cause the flooded croplands to spectrally resemble water bodies. Therefore, for applications that require high accuracy in the delineation of croplands, users are encouraged to incorporate additional data with higher temporal resolutions, using our LCLU map as the baseline map.

## Figures and Tables

**Table 1.** List of datasets whose performance in mapping settlement-related classes were compared.

Dataset	LCLU Type	Name of Settlement-Related Classes	Spatial Resolution	Year
SERVIR Mekong	Multi-class	Urban and built-up	30 m	Annually between 1987 and 2018
HBASE	Single class	Built-up and settlement	30 m	2010
GHSL	Single class	Built-up	30 m	2013–2014
Current Product	Multi-class	(1) Impervious surface, (2) villages	30 m	2016

**Table 2.** List of variables used by the random forest models and their brief descriptions. For index calculation formulas, refer to Hoffman-Hall, Loboda, Hall, Carroll, and Chen [18]. Dry–cold and dry–hot seasons refer to February–April 2016 and November 2016–January 2017, respectively, in Myanmar.

Variable	Period Covered	Data Source	Unit	Original Resolution	Description
Distance to MODIS Active Fire	2016	MODIS active fire product [31]	Meter	Vector	Euclidean distance to MODIS active fire points.
Distance to VIIRS Active Fire	2016	VIIRS active fire product [32]	Meter	Vector	Euclidean distance to VIIRS active fire points.
Distance to Roads	N/A	Updated road network (described previously)	Meter	Vector	Euclidean distance to all roadways from OpenStreetMap and our digitized road network.
Distance to 3rd Order or Greater Waterway	N/A	SRTM DEM	Meter	30 m	Euclidean distance to 3rd order or greater waterway identified using SRTM DEM following Hoffman-Hall, Loboda, Hall, Carroll, and Chen [18].
Distance to Water	N/A	North America surface water map [33]	Meter	30 m	Euclidean distance to all waterbodies.
NIR Mean Occurrence (Dry–Cold)	Dry–Cold Season	Landsat imagery	Unitless	30 m	Mean values of 3 × 3 kernels based on the NIR band of Landsat 8.
Landsat 8 Band 7 SWIR2 (Dry–Cold)	Dry–Cold Season	Landsat imagery	Unitless	30 m	Landsat 8 Band 7 (SWIR2) for the dry–cold season.
Landsat 8 Band 10 TIRS1 (Dry–Cold)	Dry–Cold Season	Landsat imagery	Kelvin	30 m	Landsat 8 Band 10 (TIRS1) for the dry–cold season.
Landsat 8 Band 11 TIRS2 (Dry–Cold)	Dry–Cold Season	Landsat imagery	Kelvin	30 m	Landsat 8 Band 11 (TIRS2) for the dry–cold season.
NBR2 (Dry–Cold)	Dry–Cold Season	Landsat imagery	Unitless	30 m	Normalized Burn Ratio 2 (NBR2 [34]) for the dry–cold season.
NDVI (Dry–Cold)	Dry–Cold Season	Landsat imagery	Unitless	30 m	NDVI for the dry–cold season.
NDWI6 (Dry–Cold)	Dry–Cold Season	Landsat imagery	Unitless	30 m	Normalized Difference Water Index using SWIR1 band (NDWI6 [35]) for the dry–cold season.
NDWI7 (Dry–Cold)	Dry–Cold Season	Landsat imagery	Unitless	30 m	Normalized Difference Water Index using SWIR2 band (NDWI7 [35]) for the dry–cold season.
Tasseled Cap Wetness (Dry–Cold)	Dry–Cold Season	Landsat imagery	Unitless	30 m	Tasseled Cap Wetness (TCW [36]) for the dry–cold season.
Texture: NIR Mean Occurrence (Dry–Hot)	Dry–Hot Season	Landsat imagery	Unitless	30 m	Mean values of 3 × 3 kernels based on the NIR band of Landsat 8.
Landsat 8 Band 7 SWIR2 (Dry–Hot)	Dry–Hot Season	Landsat imagery	Unitless	30 m	Landsat 8 Band 7 (SWIR2) for the dry–hot season.
Landsat 8 Band 10 TIRS1 (Dry–Hot)	Dry–Hot Season	Landsat imagery	Unitless	30 m	Landsat 8 Band 10 (TIRS1) for the dry–hot season.
Landsat 8 Band 11 TIRS2 (Dry–Hot)	Dry–Hot Season	Landsat imagery	Unitless	30 m	Landsat 8 Band 11 (TIRS2) for the dry–hot season.
NBR2 (Dry–Hot)	Dry–Hot Season	Landsat imagery	Unitless	30 m	NBR2 for the dry–hot season.
NDVI (Dry–Hot)	Dry–Hot Season	Landsat imagery	Unitless	30 m	NDVI for the dry–cold season.
NDWI6 (Dry–Hot)	Dry–Hot Season	Landsat imagery	Unitless	30 m	NDWI6 for the dry–cold season.
NDWI7 (Dry–Hot)	Dry–Hot Season	Landsat imagery	Unitless	30 m	NDWI7 for the dry–cold season.
Tasseled Cap Wetness (Dry–Hot)	Dry–Hot Season	Landsat imagery	Unitless	30 m	TCW for the dry–hot season.
Seasonal Difference in Tasseled Cap Wetness	2016	Landsat imagery	Unitless	30 m	Absolute value of TCW difference between the dry–hot and dry–cold seasons.
Elevation	N/A	SRTM DEM	Meter	30 m	Terrain elevation directly based on the SRTM DEM.
Slope	N/A	SRTM DEM	%	30 m	Terrain slope calculated based on the SRTM DEM.
Tree Cover	2015	Landsat VCF tree cover [25]	%	30 m	Tree cover based on Landsat VCF dataset.

**Table 3.** List of classes of the LCLU map and the corresponding input data and technical summaries.

Class	Definition	Input Data	Technical Summary
Perennial Water	Consistent water surface with low seasonal or interannual variability	GSWD [42]	Pixels mapped by GSWD as “permanent water” (water bodies with consistent extent between 1999 and 2018) or “water gain” (water bodies that emerged between 1999 and 2018).
Impervious Surface	Man-made surface such as buildings and concrete ground surface that is different from bare ground	GMIS [26]	Pixels mapped by GMIS as having impervious proportion values of larger than 1%.
Villages	Aggregation of buildings in rural areas, built on bare ground	Mapped previously	Village extent as mapped by our random forest-based village mapping algorithm.
Croplands	Croplands	GFSAD [27]	Pixels mapped by GFSAD as croplands.
Managed Forests	Forests that show signs of disturbances in the near past (i.e., since 2000)	GFC [43]	Pixels mapped by GFC as forest loss between 2000 and 2016.
Natural Forests	Forests that do not show signs of disturbance in the near past (i.e., since 2000)	Landsat VCF Tree Cover [25]	Pixels mapped by Landsat VCF product as having tree cover values of larger than 40%.
Ephemeral Water	Water surface with high seasonal or interannual variability	GSWD [42]	Pixels mapped by GSWD as “stable seasonal”, “high frequency”, “dry period”, or “wet period”. These four classes correspond to areas where high levels of seasonal or interannual variability between land and water took place.
Depressions	Areas with high levels of curvature and are likely hotspots for standing water after rain or floods	SRTM DEM	Pixels whose curvature values are between −10 and −1 and flow accumulation values are greater or equal to 3. Curvature and flow accumulation were calculated based on SRTM DEM using the corresponding tools in ArcGIS.
Bare Surfaces	Areas with limited tree cover and low NDVI values (<0.5)	GBG [29]	Pixels mapped by GBG as bare ground or have NDVI values of less than 0.5 (a threshold value chosen empirically). The NDVI values were calculated based on a Landsat cloud-free composite created for the dry-cold season in 2016 for entire Myanmar.
Shrub/Grass	Areas with limited tree cover but high NDVI values (≥0.5)	N/A	All pixels that were not mapped as the classes above were classified into this class.

**Table 4.** Results of MAX and RIGHT functions.

Map Strata	Total Samples (n)	Correct Samples Defined by MAX Function (n)	MAX (M) Function	Correct Samples Defined by RIGHT Function (n)	RIGHT (R) Function	Improvement in Accuracy when Fuzziness Is Considered (R-M)	Area Weights
Perennial water	75	44	58.67%	56	74.67%	16.00%	0.007
Impervious surface	75	34	45.33%	50	66.67%	21.33%	0.002
Villages	75	33	44.00%	63	84.00%	40.00%	0.003
Croplands	152	90	59.21%	118	77.63%	18.42%	0.190
Managed forests	75	24	32.00%	48	64.00%	32.00%	0.048
Natural forests	393	256	65.14%	332	84.48%	19.34%	0.489
Ephemeral water	75	19	25.33%	35	46.67%	21.33%	0.029
Shrub and grass	184	17	9.24%	62	33.70%	24.46%	0.229
Bare surfaces	75	23	30.67%	31	41.33%	10.67%	0.003
Total	1179	540	45.80%	795	67.43%	21.63%	
Total weighted accuracy			48.20%		69.22%	21.02%	

## Data Availability

The scripts written for this project are in Python, R, and IDL. We do not limit the access to the scripts. All scripts that were directly related to the production of this dataset have been publicly released through GitHub (https://github.com/dchengeo/Myanmar_LCLU_map/). A README file has also been uploaded through GitHub, which includes brief descriptions of the uploaded scripts. The stratified estimator design tool available on the SEPAL platform is available on sepal.io and GitHub (https://github.com/openforis/accuracy-assessment/tree/master/aa_design). The users are welcome to contact the corresponding author for the details of code implementation.
